# Supplementary material for: Learning curve and surgical outcome of robotic assisted colorectal surgery with ERAS program
Source: Sci Rep. 2022 Nov 29;12:20566. doi: 10.1038/s41598-022-24665-w (PMC9709162; doi:10.1038/s41598-022-24665-w)
Supplement: Supplementary file 2 — Supplementary Information 2. [file 41598_2022_24665_MOESM2_ESM.zip › RAL Raw data-Ñ[▒K/091/HS2_ñw╝╨░O▒Kñσ.pdf]

正式摘要(電子簽章：000000221419816)

W72 / W72 ☐ 英文標題(注意:無英文姓名-請補病人基本檔!) ☐【讀取】憑證

臺中榮民總醫院

出院病歷摘要

轉入號： 轉出號：

[redacted]

**(8)**流水編號	
**(9)**入院日期	110/07/15 (2021) 直腸外科 W72 - 072
**(10)**轉科日期	
**(11)**出院日期	110/07/22 (2021) 住院天數計 7 日

診斷

- 入院 :1. Rectal CA, cT3N1M0, stage IIIb, s/p biopsy on 2021/04/29, adenocarcinoma, MD, s/p neoadjuvant CCRT, s/p robotic assisted low anterior resection + vaginal wall tumor excision + loop ileostomy on 2021/06/10, c/w ileostomy wound infection
- 出院 :1. Rectal CA, cT3N1M0, stage IIIb, s/p biopsy on 2021/04/29, adenocarcinoma, MD, s/p neoadjuvant CCRT, s/p robotic assisted low anterior resection + vaginal wall tumor excision + loop ileostomy on 2021/06/10, c/w ileostomy wound infection s/p NPWT (2021/7/16-22)

主訴

- Poor healing of stoma wound for 3 weeks

病史

- This 51 y/o female has past history of:
  1. Rectal CA, cT3N1M0, stage IIIb, s/p biopsy on 2021/04/29, adenocarcinoma, MD, s/p neoadjuvant CCRT, s/p robotic assisted low anterior resection + vaginal wall tumor excision + loop ileostomy on 2021/06/10

  She presented with a wound nearby RLQ loop ileostomy since 3 weeks ago. She was under regular follow up at our CRS OPD, without improvement of stoma wound. Under the impression of stoma wound infection, she was admitted for further management.

體檢發現

- - General appearance :
    - easy looking , conscious clear , without cardiopulmonary distress
    - Vital signs自動帶入date 體溫(BT) 脈搏(P) 呼吸(R) 血壓(BP) 身高(BH) 體重(BW) BMI
      20210715 36C 86/min 16/min 106/64mmHg 158cm 51.8kg 20.7
    - Vital signs: B.P. 106/64 mmHg , P.R. 86 /min , R.R. 16 /min , B.T. 36 ℃
  - HEENT :
    - Eyes: no anemic conjunctiva, no icteric sclera, normal EOM
    - ENT: no otorrhea, no rhinorrhea, no erythematous mucosa, no injected tonsil, no deformity
  - Neck: supple, freely movable, trachea midline,no deformity, no engorged jugular vein , no palpable thyroid or lymph node enlargement
  - Chest:
    - Inspection: Grossly normal, symmetric and fully expansion, no accessory muscle used, no operation scar, no spider angiomata, no gynecomastia
    - Palpation: no palpable mass or nodule, no subcutaneous emphysema, no tenderness
    - Ausculation: clear breathing sound
  - Heart:
    - Percussion: normal heart size
    - Ausculation: regular heart beat, normal S1, S2, no gallop, no murmur
  - Abdomen:
    - Inspection:
      - operation scar: Loop ileostoy at RLQ abdomen, with wound infection
    - Ausculation: normoactive bowel sound, no abdominal bruits
    - palpation: soft, no muscle guarding or rigidity, no local tenderness, no rebounding pain, no palpable mass, no palpable pulsation
    - Percussion: no shifting dulllness, no hepatomegaly, no splenomagaly
  -

Peripheral pulsation :	CA	BA	RA	FA	PA	PTA	DPA
Right side :	++	++	++	++	++	++	++/+++
Left side :	++	++	++	++	++	++	++/+++

  - Genito-anus: no tenderness or palpable mass lesion or rectal shelf, normal external genitalia, normal anal tone, brown stool

  CRS CONDITION
  -
    - Lung

手術日期及方法

住院治療經過

- After admission for ileostomy wound infection, the patient was under wound care with wet dressing and then NPWT use. Empirical antibiotics was given with cefazolin(7/15-22), the patient was afebrile, with improvement of wound condition. Single contrast lower GI series was arranged on 7/22, revealing no anastomsis leakage nor colon obstruction. Under relative stable condition, the patient was discharged with OPD follow up.

合併症

一般檢查

- 累積報告 :

DATE	TIME	ALB	TP	BIL.T	BIL.D	ALKP	AST	ALT	LDH	AMY	LIPASE	NH3	Ferritin	UA
1100715	1834	3.6					19	15						

DATE	TIME	WBC	RBC	HGB	HCT	MCV	PLT	BLST	NEUT	BAND	SEG	LYM	MON	EOS	BAS
1100715	1834	5110	3.14	9.5	29.6	94.3	332		74.0			17.4	7.6	0.4	0.6

DATE	TIME	NA	K	CL	BUN	CREAT	eGFR	CA	Mg	P	CRP	ESR	PCT	D-Dimer	LACTATE
1100715	1834	139	4.1		10	0.73		8.4			0.707				

DATE	TIME	SP-GR	PH	PROT	GLUC	KETON	BILI	UROB	OB	NITR	LEU	RBC	WBC	EPCEL
1100716	1149	1.007	5.5	Negative	Negative	Negative	Negative	0.2	2+	Negative	Negative	2-5	0-2	4-6

特殊檢查

病理報告

其它

- 檢驗項目 :**Bacterial Culture(Ordinary)** 檢體:**Wound(OSTOMY)** 報告日期 :**1100719**
  附註 ：
  陰性結果：
  陽性結果： ISOLATED ORGANISM: COLONY COUNT:( )/ml
  (1) Klebsiella pneumoniae S
  (2) Escherichia coli S
  (3)
  菌落量定義： P:Pure H:Heavy M:Moderate S:Slight moderate F:Few E:Enrich

- SUSCEPTIBILITY TEST: S:Susceptible I:Intermediate R:Resistant D: Susceptible Dose Dependent (SDD) NS: Nonsusceptible

	(1)	(2)	(3)	(4)
Ampicillin				
Cefazolin	R>=64	S=4		
Clindamycin				
Clarithromycin				
Gentamicin	R>=16	R>=16		
Amikacin	S=2	S=2		
Chloramphenicol				
Ampicillin/Sulbactam	R>=32	I=16		
Penicillin				
Gentamicin(120ug)				
Trimethoprim+Sulfamethoxazole	R>=320	R>=320		
Vancomycin				
Oxacillin				
Cefpirome				
Ciprofloxacin	S=0.25	S=0.25		
Minocycline				
Carbapen (IPM)	S=1	S=0.25		
Cefoperazone/Sulbactam	S=8	S=8		
Cefotaxime				
Ceftazidime	R=16	S=0.12		
Cefepime	S=0.12	S=0.12		
Ceftriaxone	R=8	S=0.25		
Flomoxef	I=32	S=2		
Levofloxacin				
Teicoplanin				
Piperacillin/Tazobactam	S=8	S=4		
Cefmetazole				
Aztreonam				
Tigecycline				
Amoxicillin/Clavulanic acid				
Cefoxitin	R>=64	S=4		
Metronidazole				
Carbapen (MPM)				
Erythromycin				
Moxifloxacin				
Linezolid				
Ertapenem	S=0.25	S=0.12		
Colistin				
Daptomycin				

注意事項： For Enterobacteriaceae :

1.對於腸內菌屬的敏感性試驗，cefepime若採用CLSI M100-24的判讀標準，當試驗結果為敏感性時，其建議治療劑量為1gm Q12H；若以最低抑制濃度（MIC）的方法，結果為敏感劑量依賴性（susceptible-dose dependent；SDD）時，當MIC為 4 ug/mL時則建議劑量為1gm/Q8H或2gm/Q12H，若是MIC為8 ug/mL時或採用紙錠擴散法結果為SDD，則建議治療劑量為2gm/Q8H。

2.Cefazolin predict results for the oral cephalosporins agents (cefaclor, cephalexin) when used for therapy of uncomplicated UTIs due to E. coli, K. pneumoniae, and P. mirabilis.

3.Cefoperazone/Sulbactam目前並無國際性的判讀標準，現行引用文獻的判讀規範，僅供臨床參考。

出院時情況

- 3.改門診治療

出院指示

藥名	劑量	劑量單位	用法	頻次	數量	數量單位	現況	天數	單位
Cephalexin cap 500mg	1	CAP	PO	Q6H	28	CAP	TH	7	天
Strocain TAB	1	TAB	PO	TIDAC	21	TAB	TH	7	天

出院計劃

主治醫師 2062F 蔣鋒帆 / 住院醫師 2345B 陳映喆

正式摘要(電子簽章：000000221419816)

W72 / W72

doctorRank:B
